# Effects of Highly Polluted Environment on Sperm Telomere Length: A Pilot Study

**DOI:** 10.3390/ijms18081703

**Published:** 2017-08-04

**Authors:** Cecilia Vecoli, Luigi Montano, Andrea Borghini, Tiziana Notari, Antonino Guglielmino, Antonella Mercuri, Stefano Turchi, Maria Grazia Andreassi

**Affiliations:** 1 CNR Institute of Clinical Physiology, Via Moruzzi 1, 56124 Pisa, Italy; vecoli@ifc.cnr.it (C.V.); aborghini@ifc.cnr.it (A.B.); nella@ifc.cnr.it (A.M.); turchi@ifc.cnr.it (S.T.); 2 Andrology Unit of the “San Francesco d’Assisi” Hospital, ASL Salerno, EcoFoodFertility Project Coordination Unit, Oliveto Citra, 84020 Salerno, Italy; l.montano@aslsalerno.it; 3 Infertility Center, ASL Salerno Vallo della Lucania Hospital, 84078 Salerno, Italy; tiziananotari7@gmail.com; 4 Reproduction Unit, HERA Center, Sant’Agata Li Battiati, 95030 Catania, Italy; angugl@alice.it

**Keywords:** environment, telomere length, sperm quality

## Abstract

High environmental pressure may impair male fertility by affecting sperm quality, but the real effect remains controversial. Herein, we assessed the influence of environmental exposure on telomere length (TL) in both leukocytes (LTL) and sperm cells (STL). A pilot biomonitoring study was conducted in 112 clinically healthy, normospermic men living in various areas of Campania region (South of Italy) with high (*n* = 57, High Group) or low (*n* = 55, Low Group) environmental pressure. TL analysis was assessed by quantitative real time-PCR. STL was not significantly correlated with either age (*p* = 0.6) or LTL (*p* = 0.7), but was significantly longer in the High Group compared with the Low Group (*p* = 0.04). No significant difference was observed between leukocyte TL in the High or Low Group. Our results showed that male residents in areas with high environment exposure had a significant increase in STL. This finding supports the view that the human semen is a sentinel biomarker of environmental exposure.

## 1. Introduction

Male infertility has been associated with an increased risk of familial congenital malformations and childhood mortality [1,2], designating it as marker of male reproductive health. Acute and chronic exposure to high levels of ambient pollutants may impair male fertility by affecting sperm quality, but the real effect size remains controversial [3,4]. Understanding the main determinants of sperm quality and developing new biomarkers to provide a more accurate diagnosis of male fertility remains an important area of research.

In recent years, evidence has suggested that sperm DNA integrity might be a better predictor of male fertility potentiality compared to routine semen parameters [5]. However, DNA fragmentation, the most common method used in a clinical setting, seems to be unable to provide a complete information on the molecular mechanisms and severity of DNA damage [5]. Therefore, there is an urgent need for more sensitive biomarkers of DNA integrity in male germline cells.

In the last few years, more studies have focused on the role of sperm telomeres in reproduction and male infertility [6]. Importantly, the results of a recent study showed that sperm telomere length could be considered as an additional parameter of sperm quality that may add information about DNA damage and offer new perspectives in the evaluation of infertile males [7,8].

Telomere is a region of repetitive DNA at the end of a eukaryote chromosome, which protects the end of the chromosome from deterioration, conferring stability to the genome [9]. Alterations (both shortening and lengthening) of telomere length (TL) have been observed in a number of clinical conditions including cardiovascular disease and cancer [9].

Interestingly, accumulating evidence indicates that leukocyte telomeric DNA may be one important target of environmental pollutants [10,11,12,13,14,15].

Accordingly, a very recent study showed a possible association between high environmental pressure in polluted areas and sperm telomere length [16].

Herein, we performed a study on a cohort of young healthy men in order to evaluate the influence of environmental exposure on TL in both leukocytes (LTL) and sperm cells (STL).

## 2. Results

Demographic and semen characteristics of the study participants are reported in Table 1. The two groups were similar for age, body mass index (BMI), and smoking habits.

The ejaculate volume, sperm concentration, morphology, and progressive motility were not significantly different. Conversely, non-progressive motility was markedly different between the two groups (*p* = 0.005).

**Figure 1:**
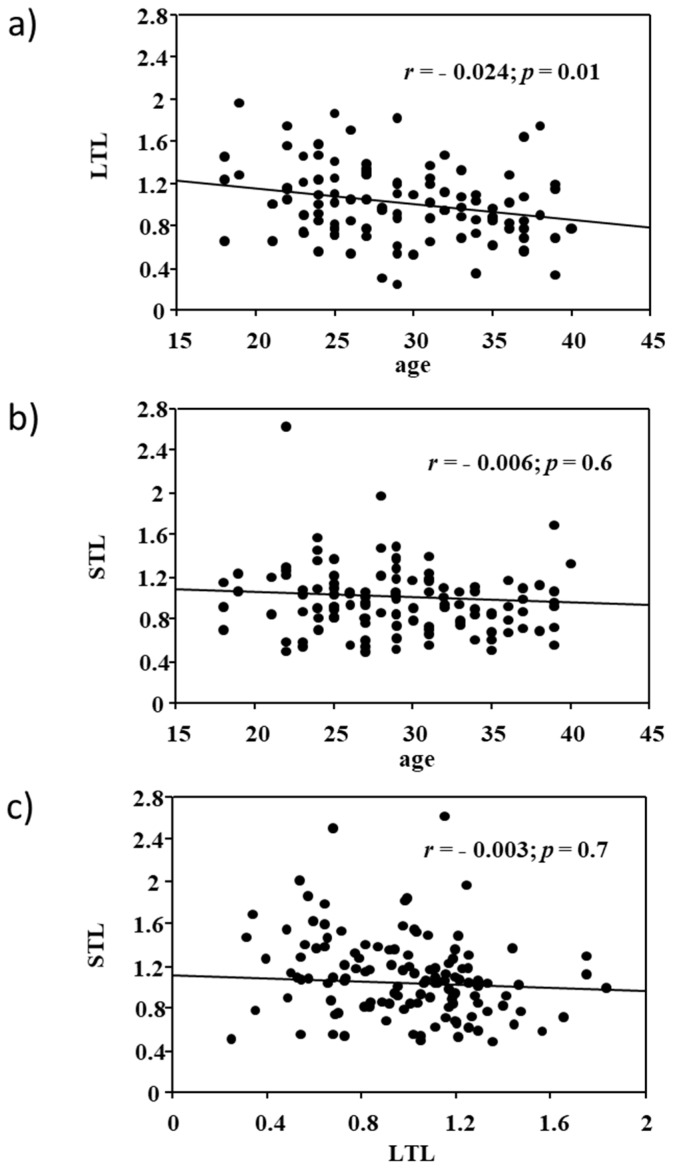
Relationship between (**a**) leucocyte telomere length (LTL) and age; (**b**) semen telomere length (STL) and age; and (**c**) LTL and STL.

**Figure 2:**
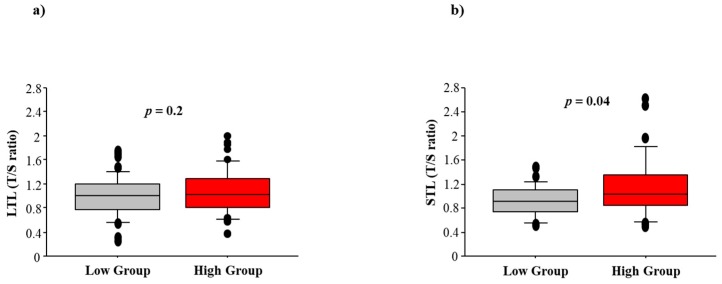
Association between high or low impact of exposure (High Group vs. Low Group) and (**a**) leucocyte telomere length (LTL) and (**b**) semen telomere length (STL).

As expected, LTL negatively correlated with subject’s age (*r* = −0.024; *p* = 0.01), but no correlation was found between STL and age (*p* = 0.6). There was also no significant association between LTL and STL (*p* = 0.7, Figure 1). No significant LTL difference was observed between the low and high exposure groups (0.99 ± 0.33 vs. 1.00 ± 0.38, *p* = 0.2). However, STL was significantly higher in the high exposure group compared to the low group (1.15 ± 0.51 vs. 0.90 ± 0.26, *p* = 0.04), as shown in Figure 2. Age-adjusted analysis confirmed a higher STL in highly exposed residents compared to the control group (1.10 ± 0.36 vs. 0.90 ± 0.32, *p* = 0.05). When STL was analyzed according to the 75th percentile distribution, living in areas with high environment exposure emerged as a significant risk predictor of longer STL (Odds Ratio: 3.1, 95% confidence intervals: 1.1–10.2; *p* = 0.02), after adjusting for age and smoking.

## 3. Discussion

The present study indicates for the first time that high levels of environmental exposure may cause an increase in semen TL of young normospermic men, supporting the major role of sperm as a sensitive sentinel biomarker of environmental effects.

### 3.1. Comparison with Other Studies

Recently, the EcoFoodFertility initiative study showed that clinically healthy adult males recruited in the “land of fire” (Campania region, Southern Italy) had altered sperm motility, and increased sperm DNA damage (DNA Fragmentation Index), when compared to subjects with similar clinical characteristics but recruited in a “low impact” area from the same region [17]. Interestingly, men residing in the high impact area had a lower total antioxidant capacity in semen but not blood [17]. Our results are consistent with and extend this previous study, showing that high environmental pressure in polluted areas may also have an influence on telomere integrity.

Telomeres, noncoding highly conserved tandem repeat DNA sequences [TTAGGG]n, are necessary for chromosome stability since they confer protection from degradation as well as prevention from chromosomal rearrangements [9]. Telomeres are elongated by the enzyme telomerase, which seems to have low activity or be totally unexpressed in most human normal somatic cells. Thus, telomeres gradually shorten in each cell division and this physiological reduction of telomere length represents a kind of biological “molecular clock” with cell senescence and longevity. Unlike somatic cells, male germline cells have a high activity of telomerase that, together with many other components including telomere-associated proteins, guarantees the integrity and stability of sperm chromosomes [6]. At the present time, the association between abnormal TL and germline cell quality represents a research area of great interest. Additionally, after fertilization, telomeres are among the first structures in the sperm nucleus that respond to oocyte signals for male pronucleus development [18]. Moreover, a long telomere is essential for a spermatozoon since it defines (jointly with the ovum) the replicative capacity until the blastocyst stage, when the telomere length is reestablished and increased. Moreover, sperm TL is lower in oligozoospermic than in normozoospermic men, and a correlation between STL and embryo quality development has been demonstrated [19]. Surprisingly, longer telomeres have been found in the spermatozoa of older men compared to younger men, but the biological implications of this paternal paradoxical effect are unknown [20]. Additionally, a relationship between dysfunctional telomere length and increased cancer risk has been observed [21]. In particular, longer telomeres have been associated with some types of cancers, including melanoma and lung cancer [22]. Indeed, long telomeres may prevent the escape of cells from growth arrest and increase the chance of acquiring mutations due to external exposures, i.e., smoking or sun exposure [22].

Importantly, a very recent Mendelian randomization study of 83 non-communicable diseases, including 420,081 cases and 1,093,105 controls, showed that longer telomeres were associated with increased risk of several cancers, but also with reduced risk of some non-neoplastic diseases, including cardiovascular diseases [23]. In this study, we found longer STL in young people living in areas of high environmental crisis where the results of the Italian Sentieri Project confirmed an increase of mortality and incidence of cancer pathologies, specifically in a young population of the “land of fire” [24].

To the best of our knowledge, only one study has previously evaluated the effect of pollutants on semen telomere length, showing a shortening in semen TL caused by an impairment in telomerase due to some types of polycyclic aromatic hydrocarbons [16].

In any case, an increase in telomere length linked to exposure has been evidenced in blood. Specifically, Shin et al. [11] found that low-dose persistent organic pollutants (POPs) increased LTL. Similarly, Dioni et al. [13] reported longer LTL associated with short-term exposure to particulate matter in a group of steel workers, likely due to an acute response of inflammatory cells. Moreover, a positive association between the level of arsenic, a known cancer-causing agent, and telomere length has been also established [14,15].

### 3.2. Study Limitations

We acknowledge that our study has several limitations that need to be considered. First, no direct ambient measures of air pollution were available. Secondly, some of the differences in the associations might be due to the relatively small sample sizes of the groups. Although two groups were homogenous for demographic and lifestyle characteristics and all analyses were adjusted for potential covariates, we cannot exclude other unidentified confounding factors.

## 4. Materials and Methods

### 4.1. Study Population

During a pilot study (EcoFoodFertility initiative) [25] conducted to investigate the use of human semen as an early biomarker of pollution in healthy men living in various areas with different environmental impact in Campania region (Southern Italy), a population of 112 clinically healthy, normospermic men (age = 29.0 ± 5.6 years, range 18–42 years) was recruited between July and December 2015 in San Francesco d’Assisi Hospital (Oliveto Citra, Province of Salerno, Italy) and Medicina Futura center (Acerra, Province of Naples, Italy). Fifty-seven subjects living in municipalities belonging to the so-called “land of fire” (Acerra, Caivano, Afragola, Casalnuovo, Pomigliano, Brusciano, Giugliano, Cardito, and Marigliano) were defined as the High Group. In this area, waste from civil, industrial, and hospital activities has been illegally dumped and in many cases burned, causing the presence of high concentrations of toxic contaminants (primarily dioxins and polychlorinated biphenyls) in the neighboring agricultural lands [26]. For this illicit practice, this area is officially recognized as having high environmental impact by the Campania Region Environmental Protection Agency [27] and is included in the list issued by the Italian Government of the 57 cities at risk within the provinces of Naples and Caserta [28].

An accurate monitoring of air quality has been provided by the local environmental control authority (namely ARPAC) since 2011 [29].

Additionally, a recent European project [30] evaluated the soil distribution patterns of heavy hydrocarbons and other polycyclic aromatic hydrocarbons in three sites of the so-called “land of fire”, demonstrating that these areas can be considered contaminated for residential use in accordance with Italian environmental law limit (Law Decree 152/2006).

Epidemiological studies performed in the last decade on the potential adverse health effects of the population living in 57 townships of the “land of fire” confirmed an increase of mortality, congenital malformations, and all types of cancer [31,32], especially in the younger population [24]. As a control group (Low Group), we enrolled 55 male residents in unexposed areas of Campania region outside the “land of fire” (Oliveto Citra, Contursi Terme, San Gregorio Magno, Buccino, Ricigliano, Valva, and Colliano).

**Figure 3:**
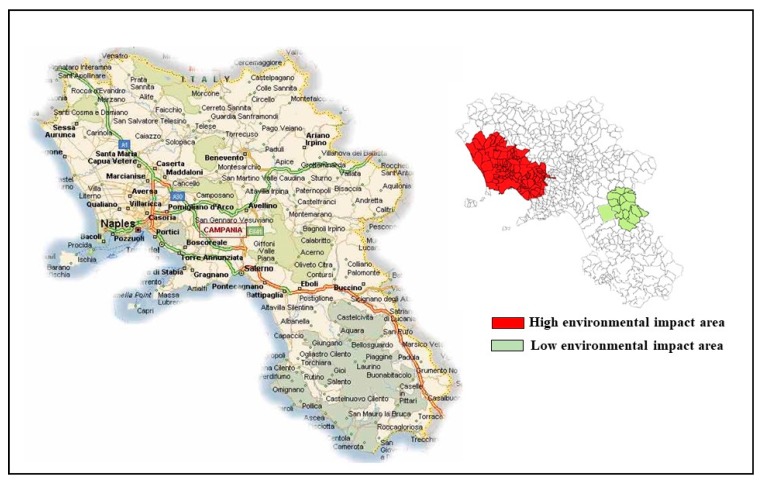
The Campania region. The map on the right side shows the geographical areas selected for the recruitment. High environmental impact areas are shown in red; low environmental impact areas are shown in green.

Figure 3 shows the geographical areas of Campania region selected for the recruitment.

Inclusion enrolment criteria were: (1) residence for at least 10 years in the study area; (2) absence of varicocele, prostatitis, and other conditions that could affect semen quality (such as fever); (3) absence of chronic diseases (diabetes or other systemic diseases); and (4) no history of drug abuse, medical exposure to X-rays, and/or occupational exposures to toxic chemicals. Data were collected by a structured questionnaire for a face-to-face interview. A physical medical examination, including the testis volume and transrectal prostate evaluation, was also performed in all enrolled subjects. All subjects were informed of the study and gave their written informed consent. The study was approved by the Local Ethical Committee (Local Health Authority Campania Sud-Salerno).

### 4.2. Semen Quality Evaluation

Ejaculates were collected upon morning masturbation after 3–4 days of abstinence. Parameters of semen quality (semen volume, sperm concentration, motility, and morphology) were evaluated by the same andrologist blinded to patient identity, according to the World Health Organization (WHO) guidelines [33].

### 4.3. Telomere Length Analysis

TL was measured quantitatively in DNA extracted from blood and semen samples (QIAamp DNA Mini Kit, Qiagen, Hilden, Germany) by the same research technician blinded to patient identity, using a quantitative real-time PCR method following standardized protocols as previously described [34]. Briefly, the relative TL (T/S ratio) was reported as the ratio of telomere repeat copy number (T) to single copy *β-globin* gene (*β-globin* gene, S). All PCRs were performed in triplicate into 384-well plates in a CFX RT-PCR System (Bio-Rad, Hercules, CA, USA).

### 4.4. Statistical Analysis

Statistical analysis of the data was performed by using the StatView statistical package, version 5.0.1 (Abacus Concepts, Berkeley, CA, USA). Data are presented as means ± standard deviation (SD). Continuous variables were first checked for normality before using parametric tests. T/S ratio was normally distributed within each study group. Characteristics of two groups were compared by χ2 test for categorical variables and by two-tailed tests, unpaired Student’s *t*-test and two-sample *t*-test, for quantitative variables.

The distribution of the sperm T/S ratio was also divided into two groups using the 75th percentile as a cut-off value (=1.18). Logistic regression was used to calculate the odds ratio and 95% confidence intervals, adjusting for age and smoking habits. The level of significance set at *p* ≤ 0.05 was considered for all statistical analyses.

## 5. Conclusions

In conclusion, the current study showed that young male residents in areas with high environment exposure had a significantly increased telomere length in sperm. These findings support the view that semen is a sensitive sentinel biomarker of environmental exposure. Further studies in larger populations are needed to understand the significance of telomere lengthening in areas of high environmental crisis.

## Figures and Tables

**Table 1:** Study population.

Variables	Low Group (*n* = 55)	High Group (*n* = 57)	*p*-Value
Age (years ± SD)	29.8 ± 5.4	28.1 ± 5.8	ns
BMI	25.2 ± 3.4	25.1 ± 3.6	ns
Smoking status, *n*	17	14	ns
Semen quality parameters			
Volume (mL)	3.1 ± 1.1	3.1 ± 1.4	ns
Cell concentration (10^6^/mL)	54.7 ± 23.6	54.5 ± 26.2	ns
Total sperm number (10^6^/ejaculate)	172.7 ± 101.9	154.2 ± 97.9	ns
Morphology (%)	6.3 ± 1.9	6.3 ± 2.0	ns
Sperm motility			
Progressive motility (%)	29.6 ± 14.3	33.0 ± 12.4	ns
Non-progressive motility (%)	18.6 ± 7.5	25.8 ± 17.2	0.005

BMI: Body Mass Index; ns: not significant.
